# Cook County Medical Society

**Published:** 1857-07

**Authors:** Thomas Bevan

**Affiliations:** Secretary


ARTICLE II.—*Cook County Medical Society.*—Meeting for June, 1857.

Society met pursuent to notice at the office of Drs. Johnson, Andrews and White.

The President, Dr. N. S. Davis, being absent, Dr. Edmund Andrews was elected President pro tem.

Minutes read and approved.

Drs. W. S. Dennison and P. J. Wardner were proposed and elected members of the Society.

Under the head of reports from committees no reports.

A paper was then read by Dr. Coatsworth, on a case of face presentation, rendered such by premature rupture of the membranes. By means of the vectis the position was changed, and the delivery completed by the vertex.

Dr. Davis in the chair.

Dr. Paoli reported a case of gun-shot wound of the chest.

Dr. Geo. K. Amerman then reported the following very interesting cases of cerebro-spinal-meningitis:

I have recently attended three cases which were to me exceedingly interesting. From the comparative infrequency of the disease, the obscurity of its true nature, and also the probability of other members having had similar cases, which will enable us to compare notes, I beg leave to present to the notice of this society a brief outline of the symptoms and treatment. For the want of a better name, as well as from a predilection in my own mind, I have called the affection "Cerebro-spinal-meningitis." The histories of these cases, from the ignorance of the parties from whom they were obtained, are imperfect and incomplete; however, I hope to be able to present them in such form as to demonstrate some of the peculiar characteristics of the affection. Besides the three cases which have come directly under my own supervision, I have heard of one other, which, from its similarity, I believe to have been of the same nature, and shall consequently give a brief account of its most prominent symptoms, which I obtained from the father subsequent to the death of the child.

I was called to the first case on Thursday, April 2d, early in the forenoon. The patient's name was Ellen Galivan, aged two years; had previously enjoyed good health, but was evidently of a scrofulous habit. From its mother I obtained the following account of its present illness: On Saturday, March 28th, she was seized with vomiting, and pain in the stomach. The matter vomited consisted apparently of the food without having underwent the slightest change, and was ejected with great ease and without nausea. The next day (Sunday) she was as well as ever, but on the day following (Monday) she was again taken with vomiting, and some febrile reaction. In the evening she was restless and fretful, and would occasionally scream from a severe cerebral pain. On Tuesday the fever increased, and the head was slightly drawn back. On Wednesday little change was observed. On Thursday, April 2d, five days after the first appearance of the affection I was called. I found her with a rapid pulse, hot skin, furred tongue, urgent thirst, and extreme restlessness. The bowels had not moved since the invasion of the attack, though several mild cathartics had been given by the mother. The urine was scanty and high colored; eyes very bright, otherwise natural; head far drawn back with inability to bring it forward; pain on pressure over the cervical vertebra or on the slightest movement of the head; at times she would rise in bed and scream from the intensity of the cerebral pain, which seemed to be paroxysmal.

I directed three leeches to be applied to the nape of the neck, a cathartic, and three grains of iod. of pot. every two hours.

On Friday. April 3d, I found my patient in nearly the same condition. She had slept none during the night, the pain and fever remaining the same as on the day previous. Bowels still constipated, and head far drawn back.

Ordered croton oil, a blister to the neck, and continued the potash.

On Saturday, Apr. 4, much improved. Febrile symptoms less active, and pain entirely disappeared. The oil produced a free movement of the bowels, which afforded great relief. Blisters drew well. The head could be brought slightly forward without producing much pain.

Continue the potash.

Monday, April 6th, still improving. No fever; good appetite; slept well last night. Head still drawn back, otherwise no evidences of the disease. This morning Prof. Davis was kind enough to see my patient and give me the benefit of his valuable opinion in regard to it. He gave a favorable prognosis, and recommended a continuation of the potash with the administration of a cathartic, which should be in part tonic as well as laxative.

Wednesday, April 8, found my patient sitting up and quite well. The head is at times thrown backward, but is easily controlled by the patient.

I directed them to use the cathartic as circumstances required, and also recommended iron and glycerine.

The second case came under my charge on Saturday, April 11, five days after dismissing the first. This was also a child named Bridget Crowly, aged 11, born in Chicago of healthy parents, and having enjoyed up to the present time uniform good health. There was no scrofulous habit or other predisposition so far as I could ascertain. The following history I obtained from her mother: On Wednesday, April 8, she assisted in taking charge of her cousin (whose case I have given below) until her death, and then remained at the "wake" on Wednesday night. She was up all night, and the next day began to feel unwell. Her strength and spirits seemed, as she expressed it, "all gone." She had lassitude and general uneasiness, with a confused mind and drowsy disposition. During the day, and soon after the first onset of uneasiness, she began to vomit. The matter vomited was of a greenish color, and seemed to be expelled without effort or nausea. In the evening the head was drawn slightly backward. She slept none during the night, but was wildly delirious, talking of her school and cousin. The next day (Friday) she remained in nearly the same condition, except a gradual increase of constitutional disturbance, and an occasional pain, with a greater tendency of the head to be thrown backward. The next day, Saturday, April 11, I was called. I found her with a rapid pulse, hot skin, coated tongue, urgent thirst, and complete anorrexia. The head was moderately drawn back, but could easily be brought forward. Eyes natural; pupils sensitive to light; bowels constipated; urine scanty. No constant pain, but at times a severe cerebral pain.

Ordered a cathartic, and blister to the neck.

On Sunday, April 12, found my patient much worse. On inquiry I found the mother had concluded the cathartic rather larger than necessary, and consequently had given only half of it, which had produced no effect. I then examined the neck, and in this also she had taken her own course. The blister, in her opinion, was too large for a child, and as one of her neighbors advised her to apply it to the nose instead of to the neck, she had compromised the matter by dividing it in two halves and applying one-half to each place. The symptoms were all aggravated, the febrile action greatly increased, the head drawn far back, and the pain at times excruciating. It was impossible to bring the head forward, but whether from spasmodic action of the post cervical muscles or from the pain occasioned by the effort I am unable to decide. There was no sensible muscular rigidity, and as the parts were painful on the slightest pressure, I am disposed to regard it as the most probable cause of the inability to flex the head.

Ordered croton oil, a small blister behind each ear, and iod. pot. in large doses. Keep the patient in a darkened and quiet room, allow her iced water to drink, and as long as grateful to her own feelings the application of cold wet cloths to her head, renewed every five minutes.

In the evening I called again, and found a decided change had taken place during the day. The oil pill, being rather small, the mother had given two instead of one (as I had directed), and the effect had been about twenty evacuations of the bowels, which had produced considerable prostration, decided decrease in the febrile symptoms, entire absence of pain in the head, and complete rationality. The head was still drawn back, and cervical spine tender on pressure. Pulse 130

and very weak; skin cool and moist; the blisters had produced no effect; no sensible effect from the potash.

Ordered them to continue the potash during the night, unless she slept, and then on no account disturb her.

Tuesday, April 14th, slightly improved; pulse 124; bowels constipated; no diuresis.

Ordered ol. ricini and terebinth. Continue the potash.

Wednesday, April 15th, improving; bowels freely moved, with the expulsion of several worms; pulse 124; head in same position; pain on pressure over the cervical vertebra, or whenever an attempt is made to flex the head.

Continue the potash.

Sunday, April 19th, head easily brought forward without pain; appetite good; sleeps well; complains of weakness.

Discontinue the potash and take quinine.

The name of my third patient was Dennis Crowley, aged 5 years, born in Chicago, brother of Bridget, and had previously enjoyed good health. During the winter he passed several worms, but had at no time been sick. On Friday, April 17th, nine days after his sister was taken, he began to feel dull and stupid. Instead of getting up that morning to take his breakfast and play, he remained in bed all forenoon, without, however, making any complaints of feeling unwell. In the afternoon he was seized with convulsions. Soon after he was visited by Dr. Gore, who administered a cathartic. Early the next morning (Saturday, April 18th), I visited him, and found he had had no vomiting, had slept some the previous night, and that his bowels were moved twice. His condition resembled a sound sleep. The limbs were flaccid, and when raised would drop as though they were perfectly useless. The eyes were partially opened and turned upward; pupils widely dilated and insensible; skin cool; pulse rapid and intermittent every fourth beat. All efforts to arouse him were perfectly useless, and his mother assured me he had been "struck deaf and dumb," which was about as plausible an explanation as I could give to account for the singularity of the symptoms. The head was slightly drawn back, but there was no cerebral or cervical pain. The absence of the vomiting, pain and fever, induced me to regard it as different from the two preceding cases, and to attribute the cerebral symptoms to intestinal irritation.

With this view I prescribed an anthelmintic.

In the afternoon he was again seized with convulsions. I was sent for, and on my arrival found him rolling and tossing about the bed, his eyes fixed and pupils contracted. Active febrile symptoms had supervened, with occasional cerebral pain; pulse rapid and intermittent; head far drawn back, with inability to bring it forward; bowels costive. This sudden and unexpected change induced me to modify my opinion, and regard the affection as one of "cerebro-spinal-meningitis," similar to the two preceding cases.

Acting in accordance with this view I directed croton oil as a cathartic, a blister to the neck, and iod. pot. in large doses.

The next morning, Sunday, April 19, his condition had not materially changed. The oil produced a free movement of the bowels, and about one dozen large long worms had been expelled, but without relieving the febrile or cerebral symptoms. The head was still drawn far back, and the child exceedingly restless. Tongue dry and coated; pulse rapid and intermittent; skin hot; urgent thirst, and urine scanty. The blister had produced no effect.

I directed the potash to be continued, and a blister behind each ear.

Monday, April 20, very slightly improved. Slept some last night; febrile symptoms somewhat less active; pulse slower and not so regularly intermittent; patient still extremely restless, with occasional twitchings of the muscles of the extremities; cervical pain on pressure; head in same position. The blisters had produced no effect; and here I would mention, that owing to the superstitious ignorance of the mother, who acted as nurse, it was impossible to have him properly cared for. Nothing was done during my absence according to directions, and the medicine was frequently omitted entirely at night, and given irregularly during the day. To this neglect, in a disease of such rapid progress as cerebro-spinal-meningitis, we may in part attribute the inefficacy of the means employed to arrest it, and the inevitably fatal termination of the case.

On Tuesday, April 21st, he was considerably worse. The muscles of the extremities were in almost constant spasmodic action, breathing labored and difficult, deglutition almost impossible. My daily notes for Wednesday and Thursday, April 22 and 23, present nothing worthy of interest. The treatment I advised was the same as in the two previous cases, but for reasons already mentioned it had no effect. The cerebral pain and fever increased, the head became far drawn back, the limbs rigid and stiff, the eyes fixed, the hands clenched, and death occurred on Friday morning, April 24, seven days after he was taken ill. No autopsy.

The fourth case was the cousin of the last two patients, and was the first of the three who suffered from this disease. I did not attend the case, but obtained from the father a brief account of the most prominent symptoms. The name of the patient was Mary O'Brien, aged 11, born in New York of healthy parents, and had always enjoyed good health. She was seized on Monday, April 6th, at 2 o'clock A. M., with vomiting. At 4 P. M. a physician was called, who administered some powders, which checked the vomiting. Monday night she had active febrile symptoms, with delirium, cerebral and cervical pain. During the next day (Tuesday, April 6), she grew rapidly worse; the fever increased, she became very delirious, and had intense cerebral pain, with the head far drawn back, bowels obstinately constipated. She died the next day, Wednesday, April 8, three days after being taken.

The clinical phenomena of the above cases present so many features in common that I do not hesitate to express my opinion that the disease was in all the same, but from the small number of cases I cannot draw any inferences or conclusions. The only object in presenting this paper was to compare notes with the observation of others who may have had similar cases, as such a comparison and collection of cases only would justify any reasoning in regard to its cause, course, pathology, or treatment.

*No.* 63 *Lake Street, Chicago.*

After the reading of the above cases, Dr. Peterson read an interesting paper on the œtiology of hemorrhoids, which will be further treated and discussed at the next meeting.

After the transaction of some miscellaneous business the Chair appointed Dr. Wickersham essayist at the next meeting, and as the subject of discussion, Cholera-infantum.

Adjourned to meet the first Tuesday in July.

THOMAS BEVAN, *Secretary.*

